# Fish oil supplementation alleviates depressant-like behaviors and modulates lipid profiles in rats exposed to chronic unpredictable mild stress

**DOI:** 10.1186/s12906-015-0778-1

**Published:** 2015-07-17

**Authors:** Mimi Tang, Pei Jiang, Huande Li, Yiping Liu, Hualin Cai, Ruili Dang, Wenye Zhu, Lingjuan Cao

**Affiliations:** Institute of Clinical Pharmacy & Pharmacology, Second Xiangya Hospital, Central South University, Changsha, Hunan 410011 PR China; School of Pharmaceutical Sciences, Central South University, Changsha, China

**Keywords:** CUMS, Fish oil, Sertraline, Lipid profiles, Adipokines, Ghrelin

## Abstract

**Background:**

Patients with major depressive disorder have a higher prevalence and incidence of dyslipidemia. However, clinical studies concerning the association between lipid levels and depression are inconsistent. Adipokines (like leptin and adiponectin) and ghrelin are strongly associated with lipid metabolism. Fish oil, which is reported to possess antidepressant effect, also have beneficial effects on lipid metabolism and the cardiovascular system. In the present study, we investigated lipid metabolism in rats exposed to chronic unpredictable mild stress (CUMS) and the effect of fish oil on lipid profiles, aforementioned adipokines and ghrelin.

**Methods:**

Sucrose preference test (SPT), open field test (OFT) and forced swimming test (FST) were used to evaluate the antidepressant-like effects of fish oil. After the behavior tests, peripheral blood were collected. Serum parameters, including fasting triglyceride (TG), total cholesterol (TCH), high density lipoprotein-cholesterol (HDL-c), low density lipoprotein-cholesterol (LDL-c), free fatty acid (FFA), glucose (GLU), adipokines (leptin, adiponectin) and ghrelin were assayed.

**Results:**

After 5 weeks of CUMS procedures, rats were induced to depressive-like state, and exhibited increased serum levels of TCH, HDL-c, FFA and decreased serum levels of leptin and ghrelin, whereas the serum status of adiponectin, GLU, TG and LDL-c remained stable. Fish oil treatment showed robust antidepressant effect and reversed the stress-induced lipid disturbance and decrease in serum concentration of ghrelin.

**Conclusions:**

Our results suggested that CUMS altered the serum levels of lipid profiles, leptin and ghrelin in rats. Fish oil supplementation not only provided antidepressant-like effects, but also reversed the altered lipid profiles and ghrelin level in serum. Our data indicated that fish oil treatment exerts anti-depressant effect and regulates lipid disturbance simultaneously.

## Background

Dyslipidemia and obesity have been observed in people with severe depression or in patients treated with tricyclic antidepressants [1], which are likely to contribute to the higher risk of cardiovascular disease (CVD). Sertraline, the selective serotonin reuptake inhibitor, is a classic and effective antidepressant and is well tolerated in patients with depression comorbid cardiovascular disease [2]. Well-documented evidence has shown that fish oil possesses antidepressant effect both on human and animals [3, 4]. Moreover, fish oil has been shown to reduce obesity and to have beneficial effects on lipid metabolism [5].

Recent clinical studies indicated that patients with depression are prone to have undesirable lipid profiles i.e. higher levels of triglyceride (TG), total cholesterol (TCH), low density lipoprotein-cholesterol (LDL-c), free fatty acid (FFA) and lower level of high density lipoprotein-cholesterol (HDL-c) than normal controls [6–8]. However, clinical studies concerning the relationship between lipid levels and depression have yielded inconsistent results [6]. To avoid the clinical interference factors, the present study was conducted in chronic unpredictable mild stress (CUMS) rats, a valid animal model of depression [9], to investigate the alteration of lipid metabolism in the affective disorder.

Recent findings have shown that adipocyte-derived hormone, leptin and adiponectin, and the gut hormone, grehlin play critical roles in energetic homeostasis, insulin sensitivity and lipid metabolism [10–12]. Interestingly, they also have important effects in the central nervous system and mood [13]. Namely, depression was highly associated with altered adipokines and ghrelin. Meanwhile, administration of exogenous adipokines or ghrelin presented an antidepressant effects [14–17]. However, the evidence concerning the interrelationship among leptin, adiponectin, ghrelin and depression is still limited and controversial [18]. Moreover, accumulating evidence suggested that fish oil administration could stimulate the serum levels of leptin, adiponectin and ghrelin [19, 20]. Therefore, the present study was carried out to investigate lipid metabolism in rats exposed to chronic unpredictable mild stress (CUMS) and the effect of fish oil on lipid profiles, aforementioned adipokines and ghrelin.

## Methods

### Animals

Male Sprague–Dawley rats, weighing 150–180 g (week 8), were initially housed in groups of three in a temperature-controlled environment under a 12-h light/dark cycle, with *ad libitum* access to food and water except during experimental procedures. All animal use procedures were conducted according to the Regulations of Experimental Animal Administration issued by the State Committee of Science and Technology of the People’s Republic of China, with the approval of the Ethics Committee in The Experimental Animal Center of the Second Xiangya Hospital.

### Drug treatment and CUMS procedures

Rats were randomly assigned to six groups (*n* = 9): (1) control, (2) control + FO, (3) control + ser, (4) CUMS, (5) CUMS + FO, (6) CUMS + ser. Fish oil (FO, EPA34 %, DHA24 %, Sheng Tianyu Biotechnology co., China) and sertraline (ser, Eastbang Pharmaceuticals, China) were dissolved in sterile saline containing 0.5 % Tween [21]. We use sertraline as positive control for behavior test of rats. The FO supplementary groups received daily gavage of 1.5 g/kg fish oil 2 weeks before the CUMS procedures started, until the end of the experiments. The sertraline groups received daily gavage of 8 mg/kg sertraline at day 1 when the CUMS procedures started [22], while the control and CUMS groups received same volume of saline containing 0.5 % Tween.

**Fig. 1:**
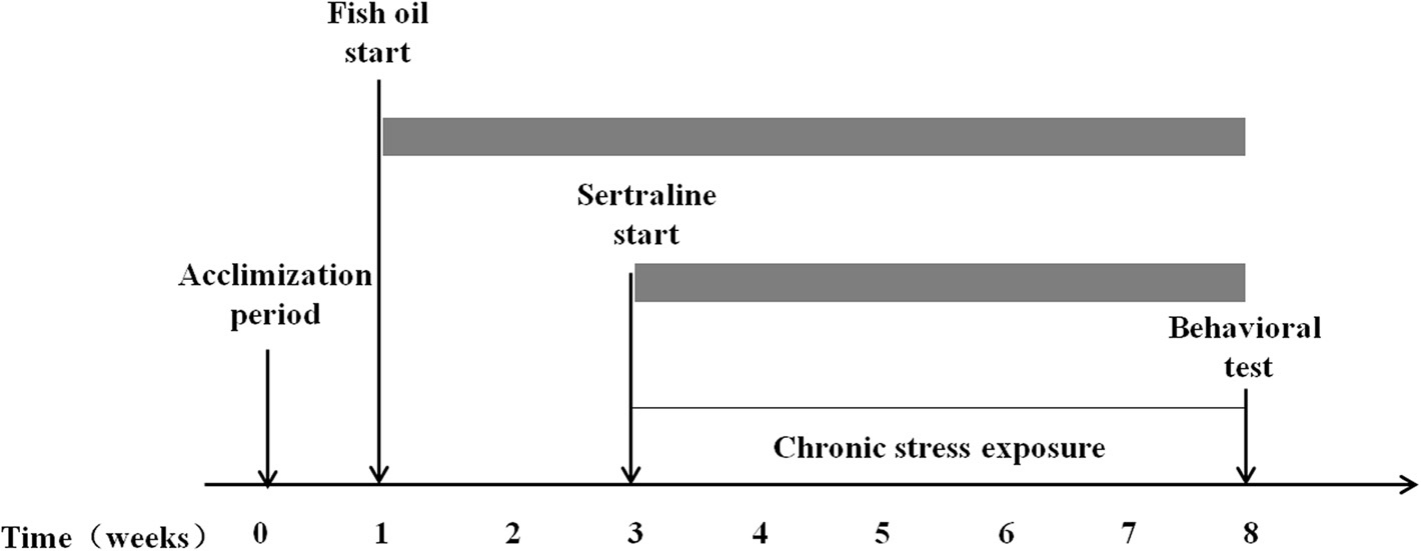
Timeline of experimental procedures

The CUMS procedures were performed as described previously with minor modifications [12]. This paradigm was designed to maximize unpredictability and mildness of the stress intensity. The rats of CUMS groups were housed in a separate cage (cage size: 26 × 19 × 15 cm), while others share one cage (three per cage, cage size: 90 × 45 × 25 cm). After acclimatized to the laboratories for 5 days, and 2 weeks gavage of fish oil or saline respectively, the CUMS groups received random unpredictable stress for 5 consecutive weeks (Fig. 1). Stress stimuli included: cage tilting for 24 h; damp bedding for 24 h; fasting for 24 h; water deprivation for 24 h, finally with 1 h an empty bottle; light–dark-cycle reversal (12 h/12 h), behavior restriction for 2 h; 30 min noise, 5 min tail pinch. Rats received one of these stressors per day and same stressor was not applied in 2 consecutive days. The rats were weighed every 3 days and the dose was adjusted to its weight gain. All animals were subjected to three behavioral tests to confirm induction of depression-like behaviors in CUMS rats as described below.

### Sucrose preference test

Anhedonia, which is a central feature of depression, was defined as a reduction in sucrose preference [23]. Briefly, rats were placed in individual cages for habituation to the sucrose solution (1 %, *w/v*): two bottles of 1 % sucrose solution were placed in the cage for the first 32 h, then after 16 h water deprivation, one bottle of 1 % sucrose solution was replaced with water for 1 h. The rats were permitted free access to the two bottles. The volumes of consumed sucrose solution and water were recorded. Completing this period, the liquid consumption was measured and the sucrose liquid drinking volume, as a percentage of the total liquid consumption, was calculated.

### Open field test

For the open field test, spontaneous locomotor activity was measured as described previously [22], with minor modifications. Reduced number of locomotor crossing and rearing reflected the loss of interest, a hallmark of depression, in rodents [24]. Briefly, the test chamber consisted of a square arena (90 cm × 90 cm × 40 cm). The floor was divided into 25 equal squares by black lines. At the beginning of the test, the animal was placed in the center of the arena and allowed to freely explore for 5 min. The apparatus was cleaned with 75 % ethanol after each testing session to prevent any odors deposited by the rats from influencing the following rat. The horizontal locomotor activities (segments crossed with all four paws) and vertical exploratory activities (standing on their hind paws) were scored.

### Forced swimming test

FST is widely used for measurement of depression-like behavior in rodents. Briefly, each rat was placed in a plastic drum (45 cm height, 25 cm diameter) containing approximately 35 cm of water (24 ± 1 °C) for a 15-min pretest. Twenty-four hours later, the rat was exposed to the same experimental conditions outlined above for a 5-min FST. Each test session was videotaped and the duration of immobility, which defined as floating passively and only making slight movements to keep the head above water, was scored by an experienced observer blind to the experiment design.

### Serum parameters

Upon completion of the experiments, all rats were weighed and blood was collected from the anesthetized animals into blood collection tubes after a 12-h overnight fast. After standing for 30 min, the serum was prepared by centrifugation of blood at 1000 × g for 10 min at 4 °C and stored at −80 °C until analysis. Serum levels of triglyceride (TG), total cholesterol (TCH), high density lipoprotein-cholesterol (HDL-c), low density lipoprotein-cholesterol (LDL-c), free fatty acid (FFA) and glucose (GLU) were measured by means of enzymatic methods, using assay kits (Sekisui Medic; Abbott Laboratories or Beijing leadman biochemistry co., LTD). Enzyme-linked immunosorbent assays were used to measure the serum levels of leptin (R&D systems, USA), ghrelin (RayBiotech, USA) and adiponectin (CUSABIO Biotech, Ltd).

### Statistical analysis

Results were expressed as mean ± SEM. Statistical analyses were performed with one-way analysis of variance (ANOVA) for multiple comparisons followed by TUKEY for post-hoc test. The prior level of significance was established at *P* < 0.05.

## Results

### Body weight gain and behavioral test

As shown in Table 1. After 5 weeks of CUMS procedures, the body weight of CUMS rats was significantly lower than the control (*P* < 0.01). Treatment of fish oil or sertraline before the establishment of CUMS model failed to restore this change. The CUMS rats showed reduced sucrose preference (*P* < 0.01) in the sucrose preference test (SPT), decreased locomotor crossing (*P* < 0.01) and rearing (*P* < 0.01) activity in the open field test (OFT) and increased immobility times (*P* < 0.05) in the forced swim test (FST). As an effective antidepressant and positive control in our study, sertraline administration improved the decreased sucrose preference in CUMS rats (*P* < 0.05). Compared with CUMS group, decreased sucrose preference was also reversed by supplementation of fish oil (*P* < 0.05). In addition, the animals in sertraline treatment group showed more numbers of crossing (*P* < 0.01) and rearing (*P* < 0.05), but fish oil treatment only had significant effect on number of crossing (*P* < 0.01). Post hoc tests indicated that fish oil (*P* < 0.01) treatment reversed the CUMS-induced decrease in immobility time.

**Table 1:** Changes in body weight and behavioral test

Groups	Body weight (g)	% Sucrose preference	OFT		Immobility time ( s )
			Number of crossing	Number of rearing	
con	357.50 ± 10.00	88.06 ± 3.24	36.71 ± 3.55	15.25 ± 2.15	62.09 ± 6.52
con + FO	361.25 ± 11.29	79.24 ± 3.87	33.21 ± 3.66	15.31 ± 2.52	56.02 + 10.28
con + ser	351.11 ± 14.28	85.42 ± 3.65	40.41 ± 3.29	14.67 ± 1.70	39.00 ± 7.64
CUMS	319.44 ± 11.74**	46.79 ± 3.35**	11.25 ± 4.46**	8.17 ± 2.22*	101.52 ± 11.43*
CUMS + FO	301.88 ± 7.32	77.01 ± 3.60^##^	35.91 ± 4.02^##^	12.64 ± 2.47	47.33 ± 9.87^##^
CUMS + ser	318.13 ± 8.76	72.46 ± 4.38^##^	37.92 ± 4.75^##^	15.63 ± 1.80^#^	55.92 ± 7.21^##^

Data are expressed as means ± SEM (*n* = 8)

*CUMS* chronic unpredictable mild stress, *con* control, *FO* fish oil, *ser* sertraline

*P < 0.05, **P < 0.01 compared to control group. ^#^ *P* < 0.05, ^##^ *P* < 0.01 compared to CUMS group

### Serum lipid profiles

**Fig. 2:**
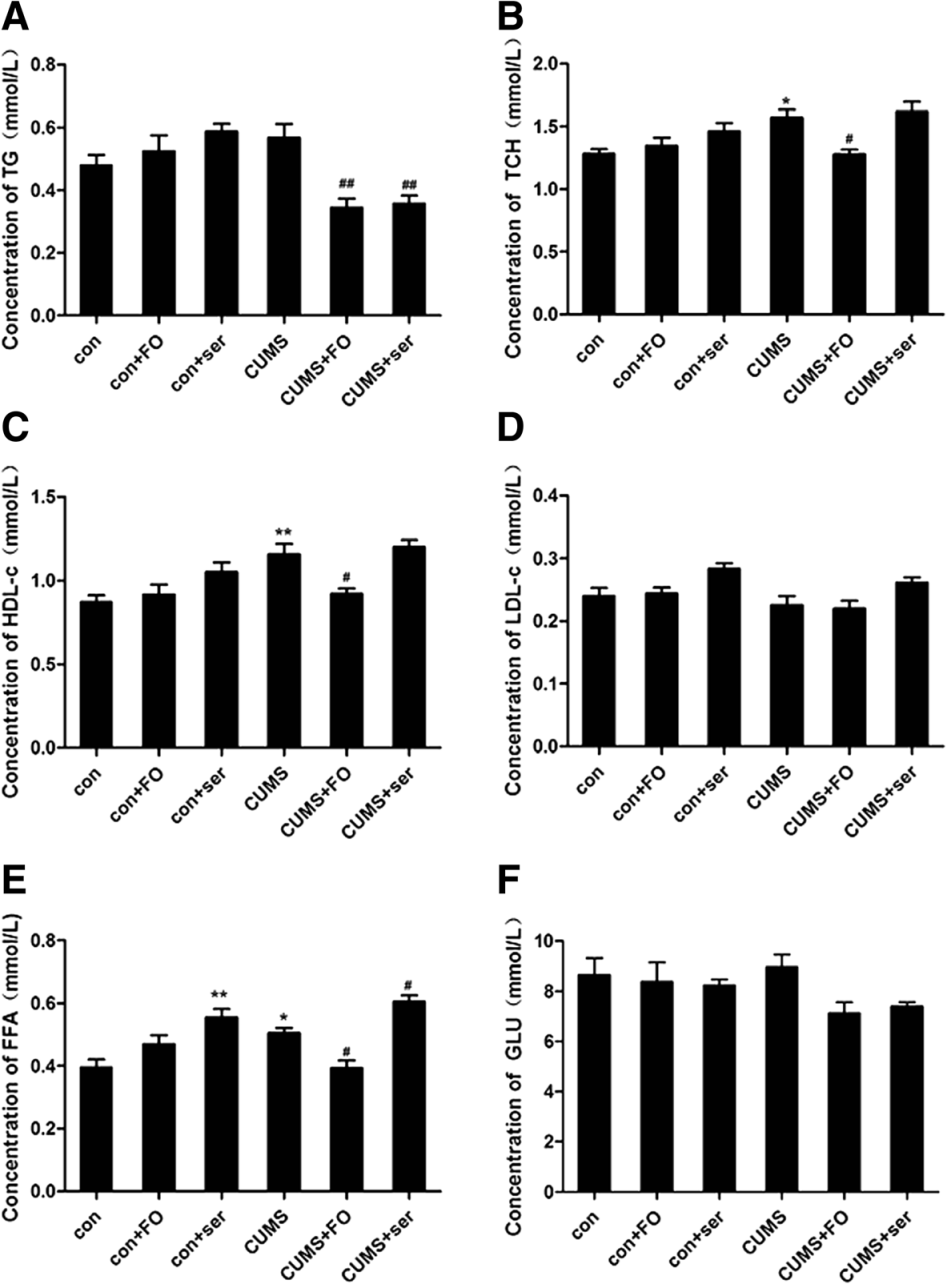
Serum levels of lipid profiles and glucose. **(A)** Concentration of TG; **(B)** Concentration of TCH; **(C)** Concentration of HDL-c; **(D)** Concentration of LDL-c; **(E)** Concentration of FFA; **(F)** Concentration of GLU. Data are expressed as means ± SEM (*n* = 8).*P < 0.05, **P < 0.01 compared to control group. ^#^ *P* < 0.05, ^##^ *P* < 0.01 compared to CUMS group

One day after the behavior tests, peripheral blood was collected. As shown in Fig. 2, CUMS significantly increased the serum levels of TCH (*P* < 0.05), HDL-c (*P* < 0.01) and FFA (*P* < 0.05), whereas TG and LDL-c remained stable. Fish oil supplementation restored CUMS-induced increase of serum TCH (*P* < 0.05) HDL-c (*P* < 0.05), FFA (*P* < 0.05), but sertraline treatment had no effect on TCH and HDL-c and signicantly increased the serum level of FFA both in control (*P* < 0.01) and CUMS (*P* < 0.05) rats. Meanwhile, the level of TG was significantly decreased by the treatment of fish oil (*P* < 0.01) and sertraline (*P* < 0.01) in CUMS rats.

### Serum levels of adipokines and ghrelin

**Fig. 3:**
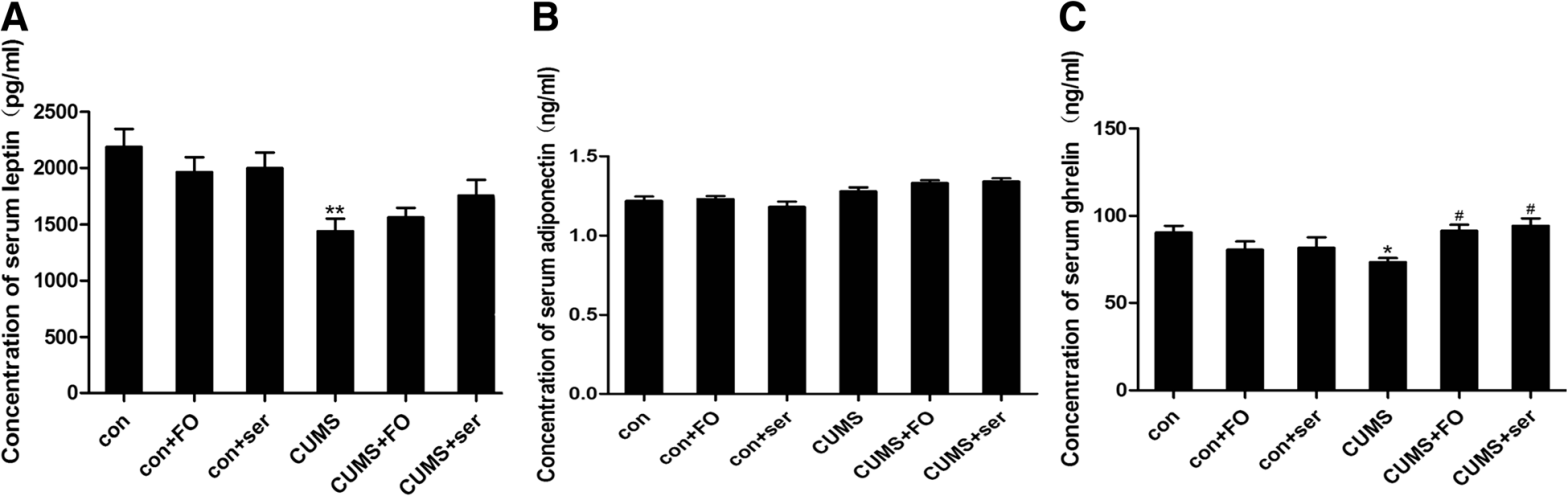
Serum levels of leptin, adiponectin and ghrelin. **(A)** Concentration of serum leptin; **(B)** Concentration of serum adiponectin; **(C)** Concentration of serum ghrelin. Data are expressed as means ± SEM (*n* = 8).*P < 0.05, **P < 0.01 compared to control group^. #^ *P* < 0.05 compared to CUMS group

As shown in Fig. 3a, the serum level of leptin was significantly down-regulated in rats that exposed to CUMS (*P* < 0.01), whereas fish oil or sertraline treatment failed to reverse this change. Meanwhile, both chronic stress and antidepressants administration had no effect on serum level of adiponectin in rats. Interestingly, we found a significant decrease of ghrelin level in CUMS rats (Fig. 3c, *P* < 0.05), whereas this reduction was restored to normal by supplementation of fish oil (*P* < 0.05) or sertraline (*P* < 0.05).

## Discussion

In the present study, CUMS, a valid model of depression for rodents, was established. The pathophysiology of depression might be related to the alterations in the lipid profiles. Animal study showed that CUMS increased plasma HDL-c significantly [25], which is different from clinical studies. Though the causes may be complicated, the discrepancy is greatly due to species specific effects. Additionally, HDLs are a class of heterogeneous lipoproteins. Different HDL subpopulations have distinct and characteristic functions, which means HDL subpopulation levels are better predictors than HDL-c levels [26]. Further studies concerning on the subpopulation levels of HDLs are needed. It has been reported that treatment with antidepressants significantly decreased both serum TCH and HDL-c levels in patients with major depression or affective disorders [6]. Elevated FFA had an adverse effect on body, causing damage to the pancreatic beta cell function, promoting cell apoptosis, and resulting in impaired glucose-stimulated insulin secretion [27]. Fish oil supplementation, an auxiliary treatment for depression, was reported to reduce the serum levels of TCH and regulate HDL-c composition and metabolism [28, 29]. In parallel with previous evidence, CUMS increased the serum levels of TCH, HDL-c and FFA, which reflected the dyslipidemia in CUMS rats. Interestingly, fish oil supplementation reversed these changes and improved the dysfunctional of lipids metabolism. On the contrary, sertraline administration failed to reverse the serum levels of TCH and HDL-c and further elevated serum concentration of FFA. It’s worth mentioning that diabetes mellitus is now perceived as an important comorbid condition in patients with depression [30]. Stress-induced increase in FFA level may partly explain the comorbid depression and diabetes. There is considerable evidence suggests that TG play a role in adverse health conditions like metabolic syndrome (MetS) [31]. Fish oil was found to reduce the serum level of TG level both in human and mice [29, 32]. Similar to the previous study, fish oil or sertraline administration in CUMS groups decreased the serum level of TG. We have observed that excessive fish oil intake may significantly lower the serum lipids, and prevent the disturbance of lipid metabolism, but the effect of sertraline on lipid regulation is mild and even deteriorated. Fish oil might be an excellent replacement therapy in depression for its beneficial effects on the progression of both depression and dyslipidemia.

Adipokines, like leptin and adiponectin, are well known for their roles in energy homeostasis [12]. Recently, accumulated studies have demonstrated the negative relationships between leptin level and severity of symptoms of depression in human [33]. Animal studies also showed that various chronic stress [15, 33–35] significantly decreased the serum concentration of leptin. Although leptin insufficiency might be associated with depression-like behavior, and leptin had antidepressant-like efficacy in rats exposed to chronic stress [15], most clinical data indicated that antidepressant medications, such as fluxetine, imipramine, paroxetine and venlafaxine had no effect on leptin level [16]. In the present study, the serum level of leptin was significantly down-regulated in rats exposed to CUMS. Similar to the previous studies using various antidepressants, fish oil or sertraline supplementation also failed to reverse leptin level. Chronic social-defeat stress was reported to reduce the plasma level of adiponectin, and administration of exogenous adiponectin produced antidepressant-like behavioral effects in mice [14]. Some reports showed higher serum concentration of adiponectin in MDD subjects compared to healthy controls [36], while other investigators found either lower levels [11, 37] or unaltered [38, 39] peripheral adiponectin levels in MDD individuals versus controls. In the present study, both chronic stress and antidepressants supplementary had no effect on serum concentration of adiponectin in rats. Ghrelin, a gut hormone, had an orexigenic effect on appetite and energy balance. Recent study suggested that serum level of ghrelin was decreased in patients with depression [16]. A subgroup of healthy volunteers who received a single intravenous injection of ghrelin after an overnight fast developed elevated mood [17]. Activation of ghrelin signaling was also reported to produce antidepressant-like response in mice [40]. In the present study, we found a significant decrease of ghrelin level in CUMS rats, whereas this reduction was restored by supplementation of fish oil or sertraline. These findings indicated that decreased leptin and ghrelin levels might show an important role in the chronic stress-induced depression. Fish oil treatment might play an antidepressant effect through increasing the serum concentration of ghrelin, the similar mechanism to sertraline, providing a potential target for treatment of depression.

## Conclusions

The main novel finding of the present study were as follows: 1) chronic stress could alter the lipid profiles, leptin and ghrelin of rats; 2) treatment with fish oil not only provided antidepressant-like effects, but also reversed disturbance of lipid metabolism and restored the serum status of ghrelin in CUMS rats, whereas sertraline could only reverse the alerted ghrelin. Since sertraline have mild effect on or even aggravate the dyslipidemia in CUMS rats, which contributes to the higher risk of CVD, fish oil treatment might be an effective auxiliary treatment for patients with depression.

## Abbreviations

ANOVA: Analysis of variance
CUMS: Chronic unpredictable mild stress
CVD: Cardiovascular disease
CHD: Coronary heart disease
ECT: Electroconvulsive therapy
FFA: Free fatty acid
FO: Fish oil
FST: Forced swimming test
GLU: Glucose
HDL-c: High density lipoprotein-cholesterol
LDL-c: Low density lipoprotein-cholesterol
MetS: Metabolic syndrome
OFT: Open field test
ser: Sertraline
SPT: Sucrose preference test
TG: Triglyceride
TCH: Total cholesterol
